# Supplementary material for: Kinesin-14 motors drive a right-handed helical motion of antiparallel microtubules around each other
Source: Nat Commun. 2020 May 22;11:2565. doi: 10.1038/s41467-020-16328-z (PMC7244531; doi:10.1038/s41467-020-16328-z)
Supplement: Supplementary file 1 — Supplementary Information [file 41467_2020_16328_MOESM1_ESM.pdf]

Supplementary Information

Mitra et al., **The kinesin-14 (Ncd) drives a right-handed, helical motion of antiparallel microtubules around each other**

Supplementary Figures

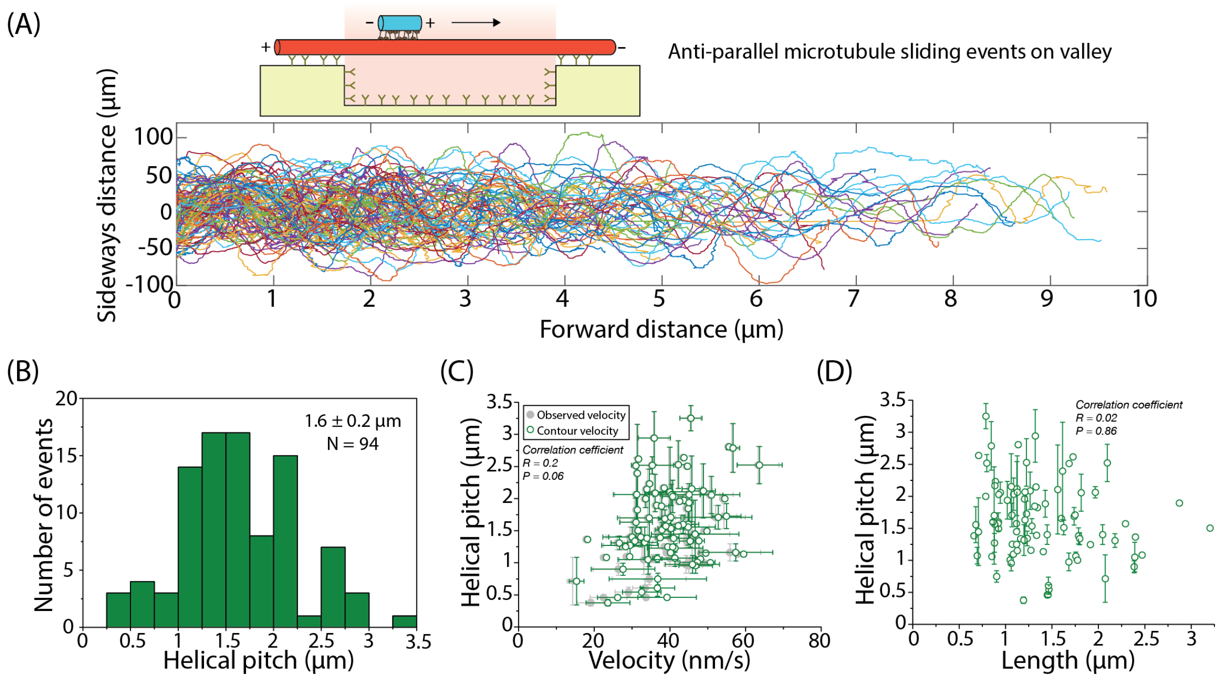

Supplementary Figure 1: Transport microtubules exhibit variable helical pitches, independent of sliding velocity and microtubule length. (A) Sideways distance (rolling-frame averaged over 20 frames) of 94 antiparallel transport microtubules moving along the suspended parts of template microtubules. Each transport microtubule exhibited a robust helical motion with fairly constant helical pitch. Between various transport microtubules, helical pitches varied between 0.5 µm and 3 µm. **(B)** Histogram of helical pitches showing a median helical pitch of 1.6 ± 0.2 µm (see methods for analysis of distributions with error estimation). **(C)** Helical pitch (mean ± SD) plotted with respect to the contour velocity (mean ± SD) of the sliding events. The contour velocity was calculated from the observed velocity (also plotted in the background in grey) accounting for the fact that the transport microtubules moved along a helical path around template microtubules. The actual (contour) velocity is therefore slightly higher than the observed velocity. There is no evident dependence of the helical pitch on the sliding velocity (R = 0.2, P = 0.06; two-sided Pearson correlation coefficient). **(D)** Helical pitch (mean ± SD) plotted with respect to the length of the sliding template microtubules. Smaller template microtubules showed slightly larger variations in helical pitch but no correlation with microtubule length was observed (R = 0.02, P = 0.86; two-sided Pearson correlation coefficient). N = 94 events for the data shown in the figure.

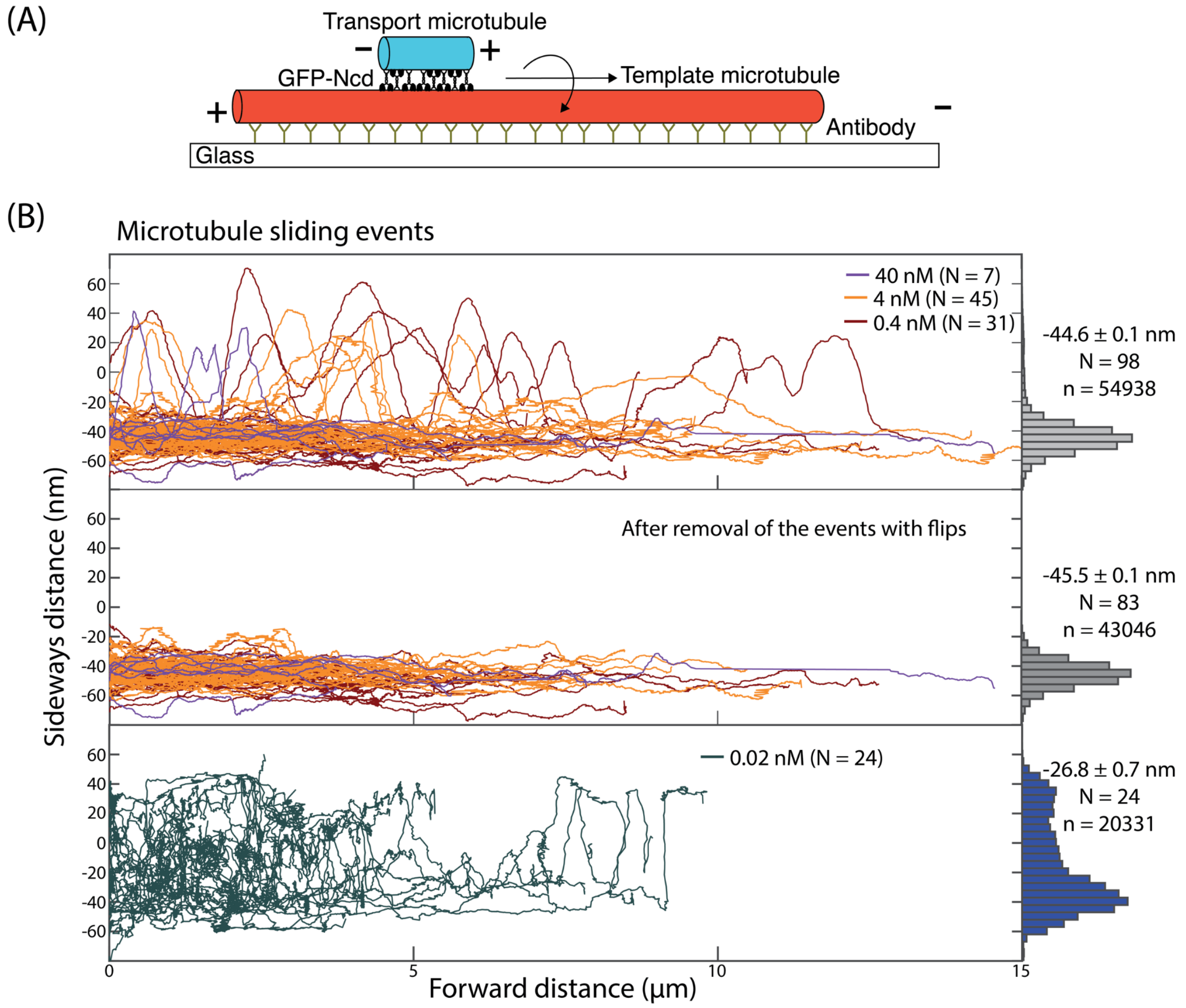

Supplementary Figure 2: The 3D motion of transport microtubules is hindered in 2D sliding motility assays with surface-immobilized template microtubules. (A) Schematic representation of Ncd-driven sliding of an Atto647n-labeled transport microtubule along a surface-immobilized, rhodamine-labeled, template microtubule. **(B)** Sideways distance of antiparallel transport microtubules at different Ncd motor concentrations. At 0.4 nM, 4 nM and 40 nM Ncd motor concentrations (upper panel), most transport microtubules slid on the right-hand side of the template microtubule (see example sliding event at 4 nM Ncd motor concentration in Supplementary Movie 2). Some transport microtubules (15 out of 98) squeezed underneath the template microtubules and performed complete flips. The mean sideways distance was -44.6 ± 0.1 nm (N = 98 events). When considering only the sliding events which remained on the right-hand side of the template microtubule, the average sideways distance was -45.5 ± 0.1 nm (N = 83 events). At 0.02 nM Ncd motor concentration the transport microtubules no longer remained on the right-hand side of the template microtubule but moved around erratically (in axial direction). There was still a slight preference for the right-hand side as indicated by the average sideways distance (-26.8 ± 0.7 nm; N = 24 events). In all panels the displayed trajectories were smoothened (rolling frame averaged over 20 frames).

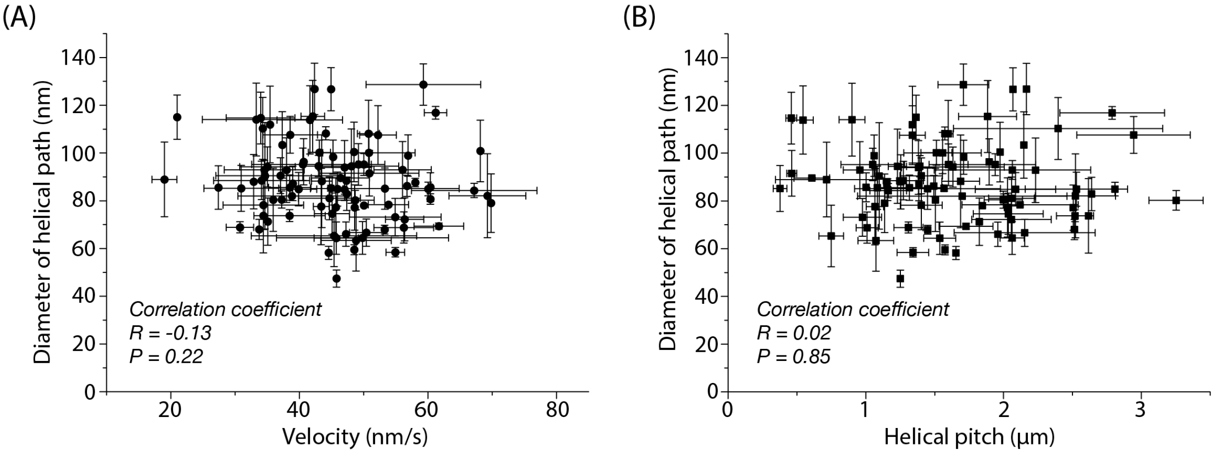

Supplementary Figure 3: The spatial extension of Ncd motors between sliding microtubules is independent of sliding velocity and helical pitch. (A) Diameter of the helical path taken by antiparallel transport microtubules around suspended template microtubules (mean ± SD) plotted in dependence of sliding velocity (mean ± SD). There was no correlation of the *in-situ* extension of Ncd motors with the microtubule sliding velocity (R = -0.13, P = 0.22; two-sided Pearson correlation coefficient). **(B)** Diameter of the helical path taken by antiparallel transport microtubules around suspended template microtubules (mean ± SD) plotted in dependence of the helical pitch (mean ± SD). There was no correlation of the *in-situ* extension of Ncd motors with the helical pitch (R = 0.02, P = 0.85; two-sided Pearson correlation coefficient). N = 94 events for the data shown in the figure.

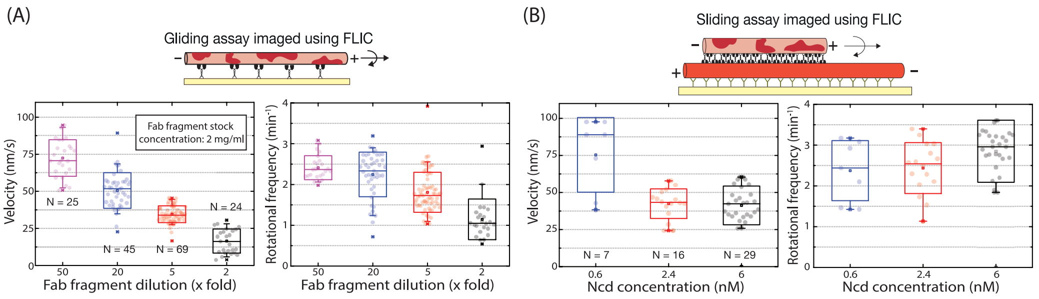

(C)

Gliding

Fab fragment dilution		p-values: pitch	velocity	frequency
50x	20x	1.9×10^{-5}	3.2×10^{-8}	0.5897
50x	5x	6.8×10^{-12}	1.6×10^{-13}	9.3×10^{-8}
50x	2x	3.9×10^{-9}	2.1×10^{-9}	3.6×10^{-8}
20x	5x	3.5×10^{-3}	6.8×10^{-13}	9.5×10^{-6}
20x	2x	1.8×10^{-5}	2.5×10^{-11}	2.0×10^{-8}
5x	2x	6.7×10^{-4}	3.9×10^{-12}	2.3×10^{-8}

Sliding

Ncd concentration		p-values: pitch	velocity	frequency
0.6 nM	2.4 nM	2.1×10^{-5}	0.0211	0.8673
0.6 nM	6 nM	5.4×10^{-5}	0.0045	0.0786
2.4 nM	6 nM	0.0235	0.8218	0.0235

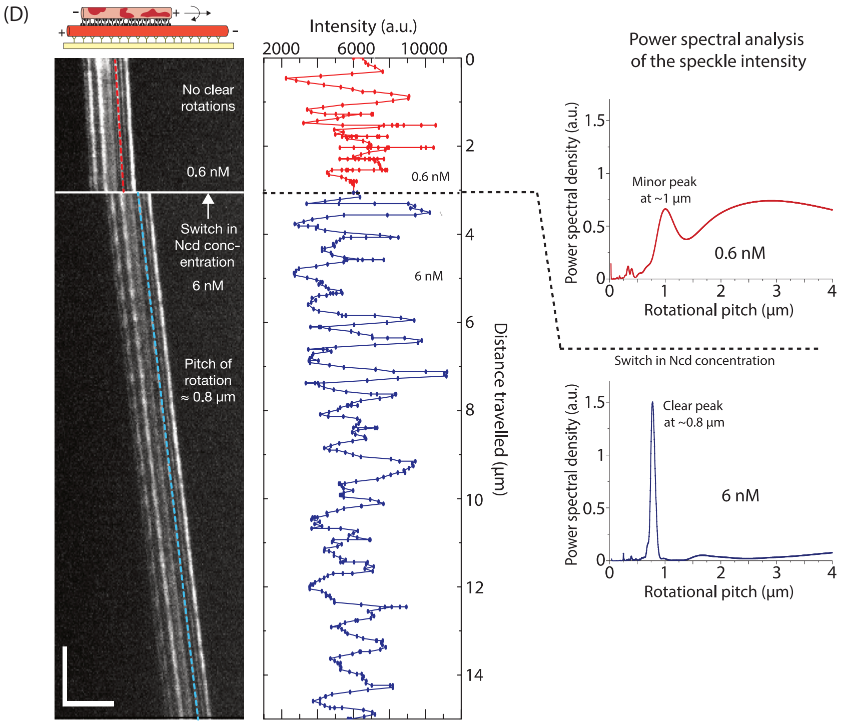

Supplementary Figure 4: Details on fluorescence interference contrast (FLIC) based gliding and sliding motility assays. (A) Box plots showing that gliding velocity and rotational frequency of microtubules gliding on Ncd motors are dependent on motor density. The rotational frequency saturates at 2 - 2.5 rotations per minute at low motor densities. The motor concentration was set by changing the Fab fragment concentration (stock concentration 2 mg/ml; diluted 50x, 20x, 5x and 2x; N = 25, 45, 69 and 24 events, respectively). **(B)** Box plots showing that sliding velocity and rotational frequency of transport microtubules sliding on surface-immobilized template microtubules were slightly dependent on the motor concentration (0.6 nM, 2.4 nM and 6 nM; N = 7, 16 and 29 events, respectively). The rotational frequency ranged between 2.5 - 3 rotations per minute. **(C)** Tables providing an overview of p-values obtained from two-sample two-sided Mann-Whitney-U-tests, corresponding to all motor concentrations in the gliding and sliding motility assays. Red boxes indicate the concentrations where the observed parameter is not significant. **(D)** Example kymograph, FLIC intensity profiles over time for one of the speckles indicated in the kymograph and the corresponding power spectral analysis, when the concentration of Ncd motors was increased to 6 nM during imaging (horizontal scale bar: 40 s; vertical scale bar: 10 µm). At 0.6 nM Ncd the microtubule did not show any clear rotational motion. However, after switching to 6 nM Ncd the microtubule rotated robustly around its own axis with a rotational pitch of about 0.8 µm. For the boxplot description in (A) and (B), see Methods.

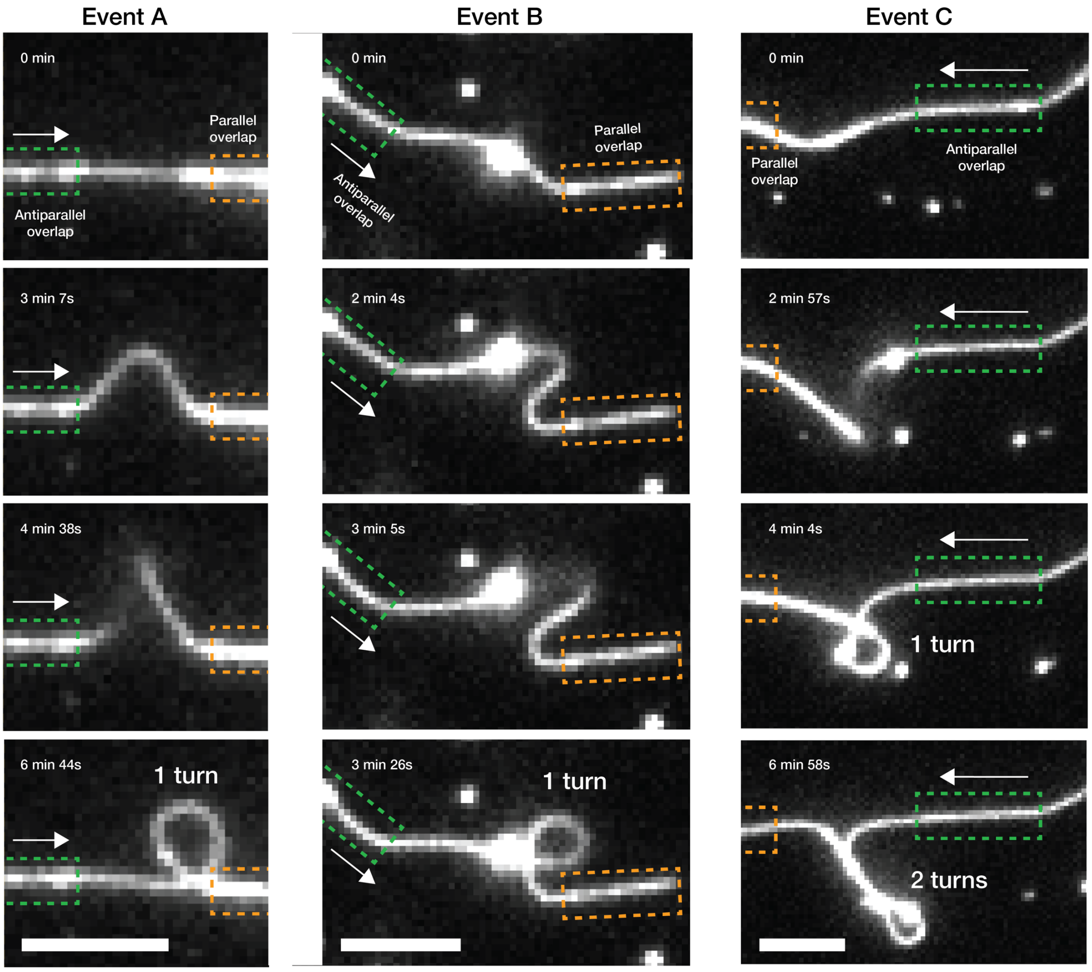

Supplementary Figure 5: Additional example events illustrating the twisting of Ncd-driven, sliding transport microtubules. Time-lapse micrographs of three example transport microtubules (events A, B and C) which have their leading end locked in a parallel configuration on one template microtubule while the trailing end is sliding in an antiparallel configuration on another template microtubule. The rotational motion of the transport microtubule around its own axis induces the central region of the microtubule to twist and eventually bend into a loop. The positions of parallel and antiparallel microtubule overlaps are indicated by the orange and green boxes, respectively. Scale bar 5 µm.
