# Supplementary material for: Kinesin-14 motors drive a right-handed helical motion of antiparallel microtubules around each other
Source: Nat Commun. 2020 May 22;11:2565. doi: 10.1038/s41467-020-16328-z (PMC7244531; doi:10.1038/s41467-020-16328-z)
Supplement: Supplementary file 3 — Description of Additional Supplementary Files [file 41467_2020_16328_MOESM3_ESM.pdf]

Description of Additional Supplementary Files

File Name: Supplementary Movie 1
Description: Example Atto647n-labeled transport microtubule (cyan) sliding along a rhodamine-labeled template microtubule (white) suspended over two valleys (10 µm wide) with a ridge (2 µm wide) in between, at 4 nM Ncd concentration. The averaged tracked position of the template microtubule is indicated by the red line and the trajectory corresponding to the transport microtubule is indicated by the blue line. Details of this event are also shown in Fig. 1C.

File Name: Supplementary Movie 2
Description: Example Atto647n-labeled transport microtubule (cyan) sliding along a rhodamine-labeled template microtubule (white) immobilized on a glass coverslip, at 4 nM Ncd concentration. The averaged tracked position of the template microtubule is indicated by the red line and the trajectory corresponding to the transport microtubule is indicated by the blue line.

File Name: Supplementary Movie 3
Description: Example rhodamine-speckled transport microtubule (cyan) sliding along a Cy5-labeled template microtubule (red) immobilized on a reflective silicon wafer, at 6 nM Ncd concentration. The kymograph corresponding to this event is shown in Fig. 4C.

File Name: Supplementary Movie 4
Description: Example Atto647n-labeled transport microtubule (blue) locked at the leading-end to a parallel rhodamine-labeled template microtubule (red) and sliding at the trailing-end along an antiparallel rhodamine-labeled template microtubule (red) on a glass coverslip, at 4 nM Ncd concentration. The rotational motion twists the central part of the transport microtubule and further causes it to bend into a loop, before coiling up into a double helix (with the loop becoming smaller and the straight part of the coiled-coil growing longer over time). We note, that the movie acquired on the camera chip is equivalent to viewing from the top onto the surface, as illustrated in Fig. 5A. When bending away from the surface, the microtubule progressively gets out of focus. A time-lapse micrograph corresponding to this event is shown in Fig. 5B. Scale bar: 10 µm; time stamp: min:sec.
